# Next generation sequencing is a highly reliable method to analyze exon 7 deletion of survival motor neuron 1 (*SMN1*) gene

**DOI:** 10.1038/s41598-021-04325-1

**Published:** 2022-01-07

**Authors:** Sumin Zhao, Yaoshen Wang, Xiuqing Xin, Zhonghai Fang, Linlin Fan, Zhiyu Peng, Rui Han, Chaonan Shi, Yixiang Zhang, Chuang Fan, Jun Sun, Xuelian He

**Affiliations:** 1 Tianjin Medical Laboratory, BGI-Tianjin, BGI-Shenzhen, Central Avenue 55, Airport Business Park East Building E3, Tianjin, 300308 China; 2 BGI Genomics, BGI-Shenzhen, Bei Shan Industrial Zone, Yantian District, Shenzhen, 518083 Guangdong China; 3 Precision Medical Center, Wuhan Children’s Hospital (Wuhan Maternal and Child Healthcare Hospital), Tongji Medical College, Huazhong University of Science & Technology, Wuhan, China; 4 Ashbury College, 362 Mariposa Avenue, Rockcliffe Park, ON Canada

**Keywords:** Genetics, Diseases

## Abstract

Spinal muscular atrophy (SMA) is one of the most common and severe genetic diseases. SMA carrier screening is an effective way to identify couples at risk of having affected children. Next-generation sequencing (NGS)-based expanded carrier screening could detect *SMN1* gene copy number without extra experiment and with high cost performance. However, its performance has not been fully evaluated. Here we conducted a systematic comparative study to evaluate the performance of three common methods. 478 samples were analyzed with multiplex ligation probe amplification (MLPA), real-time quantitative polymerase chain reaction (qPCR) and NGS, simultaneously. Taking MLPA-based results as the reference, for 0 copy, 1 copy and ≥ 2 copy *SMN1* analysis with NGS, the sensitivity, specificity and precision were all 100%. Using qPCR method, the sensitivity was 100%, 97.52% and 94.30%, respectively; 98.63%, 95.48% and 100% for specificity; and 72.72%, 88.72% and 100% for precision. NGS repeatability was higher than that of qPCR. Moreover, among three methods, NGS had the lowest retest rate. Thus, NGS is a relatively more reliable method for *SMN1* gene copy number detection. In expanded carrier screening, compared with the combination of multiple methods, NGS method could reduce the test cost and simplify the screening process.

## Introduction

Spinal muscular atrophy (SMA) is a common neuromuscular disorder. It is attributed to the degeneration of spinal and brainstem motor neurons, leading to progressive muscle weakness and atrophy and even respiratory failure and death. Its incidence is approximately 1 in 10,000 live births^1–5^. SMA is divided into four clinical subtypes according to the onset age and clinical manifestations^3^. All these forms are caused by mutations in the survival motor neuron 1 (*SMN1*) gene. More than 95% of SMA cases are caused by exon 7 deletion of this gene^6^. Additionally, *SMN2* is a highly homologous gene to *SMN1*, with only several base pair differences^7^. A C-to-T nucleotide transition in exon 7 of *SMN2* gene (c.840C>T) results in SMN2 protein truncation and instability through its effects on the exonic splicing enhancer, which is considered a critical functional consequence and distinguishes *SMN1* from *SMN2* gene. Many molecular genetic methods have been developed based on these differentiated nucleotides for *SMN1* gene mutations detection.

SMA is a fatal disease, and the treatments are expensive. Carrier screening aims at identifying carrier couples with the risk of having affected babies of inheriting recessive disorders so as to provide genetic procreation choices and early medical management combined with genetic counseling. In 2008, the American College of Medical Genetics (ACMG) recommended that SMA carrier screening should be offered to all the couples^6^. In the guidelines for genetic condition carrier screening provided by the American College of Obstetrics and Gynecology (ACOG), SMA was one of the disorders recommended for carrier screening for all the women^8^. It is of great significance to prevent the births of the affected births^9–11^. The frequently used methods for SMA carrier screening are real-time quantitative polymerase chain reaction (qPCR)^11–14^, denaturing high-performance liquid chromatography (DHPLC)^9^, and multiplex ligation probe amplification (MLPA)^15–17^. Among them, MLPA is considered the gold standard technique, and qPCR is one of the most commonly used methods. Next-generation sequencing (NGS) method has been reported in recent years for SMA carrier screening^18–20^. Especially for expanded carrier screening (ECS) including SMA with NGS, extra molecular tests no longer have to be supplemented to detect SMA, simplifying the detection process and reducing the detection cost.

The carrier rate of SMA in different ethnic groups is variable, ranging from approximately 1% to 3%^13,16,17^, which may be caused by ethnic difference. However, for individuals from the same ethnic group, SMA carrier rate varied widely between the studies. For examples, SMA carrier rate had been reported as 1.43%^17^ and 1.88%^13^ in Southeast Asian, respectively. It ranges from 0.85 to 1.46% for African American^13,14,17,21^. These differences may be related to the sample size of different studies. However, it cannot be ruled out the possibility that the accuracy of the methods used may cause such differences. In a newborn screening study of SMA with real-time polymerase chain reaction, 8 of 15 positive results were confirmed as false-positives^22^. What’s more, the sensitivity and specificity of PCR-based methods were repeatedly reported to be less than 100%^12,23,24^. The advantages and limitations of the technical methods have been summarized, including qPCR, MLPA and DNA sequencing, et al.^25^. However, a comprehensive performance analysis and comparison has not been reported. In this study, we selected the frequently used qPCR method and the promising NGS approach and investigated their performance systematically for SMA detection. These methods were carried out in accordance with relevant guidelines and regulations.

## Results

### Study design

A total of 478 samples were re-analyzed with MLPA, qPCR and NGS methods simultaneously in this study. The MLPA results were used as the reference for the validation of qPCR and NGS methods. Prior to this, the samples with failed detection or ambiguous copy number results by MLPA were redetected until the effective copy number was identified (0, 1, 2, 3 or 4 copies). Additionally, 71 of the 478 samples, namely, 15 *SMN1* homozygous deletion samples, 27 *SMN1* heterozygous deletion samples and 29 *SMN1* nondeletion (≥ 2 copies) samples, were selected for the repeatability study of NGS and qPCR methods.

### Sensitivity and specificity

With MLPA method, 16 of the 478 samples were classified as homozygous deletion (0 copy of *SMN1*), 133 as heterozygous deletion (1 copy of *SMN1*), and 329 as normal (≥ 2 copies of *SMN1*); these results were consistent with the previous molecular diagnostic results. Taking MLPA-based results as the reference, in the qPCR experiments, the sensitivity, specificity and precision for the detection of 0 copy *SMN1* gene were 100%, 98.63% and 72.72%, respectively. They were 97.52%, 95.48% and 88.72%, respectively for 1 copy *SMN1* gene identification, and 94.30%, 100% and 100% for ≥ 2 copy *SMN1* analysis (Table 1). In the NGS experiments, the sensitivity, specificity, and precision were all 100% for the analysis of *SMN1* gene copy number (Table 2).

**Table 1:** The performance of qPCR method for *SMN1* copy number detection.

MLPA results		qPCR results						
		Number of samples with 0 copy *SMN1* (n)	Number of samples with 1 copy *SMN1* (n)	Number of samples with ≥ 2 copy *SMN1* (n)	Number of samples with failed or ambiguous results (n)	Sensitivity (%)	Specificity (%)	Precision (%)
Number of samples with 0 copy *SMN1*	16	16	0	0	0	100	98.63	72.72
Number of samples with 1 copy *SMN1*	133	3	118	0	12	97.52	95.48	88.72
Number of samples with ≥ 2 copy *SMN1*	329	3	15	298	13	94.30	100	100

**Table 2:** The performance of NGS method for *SMN1* copy number detection.

MLPA results		NGS results						
		Number of samples with 0 copy *SMN1* (n)	Number of samples with 1 copy *SMN1* (n)	Number of samples with ≥ 2 copy *SMN1* (n)	Number of samples with failed or ambiguous results (n)	Sensitivity (%)	Specificity (%)	Precision (%)
Number of samples with 0 copy *SMN1*	16	16	0	0	0	100	100	100
Number of samples with 1 copy *SMN1*	133	0	128	0	5	100	100	100
Number of samples with ≥ 2 copy *SMN1*	329	0	0	321	8	100	100	100

**Figure 1:**
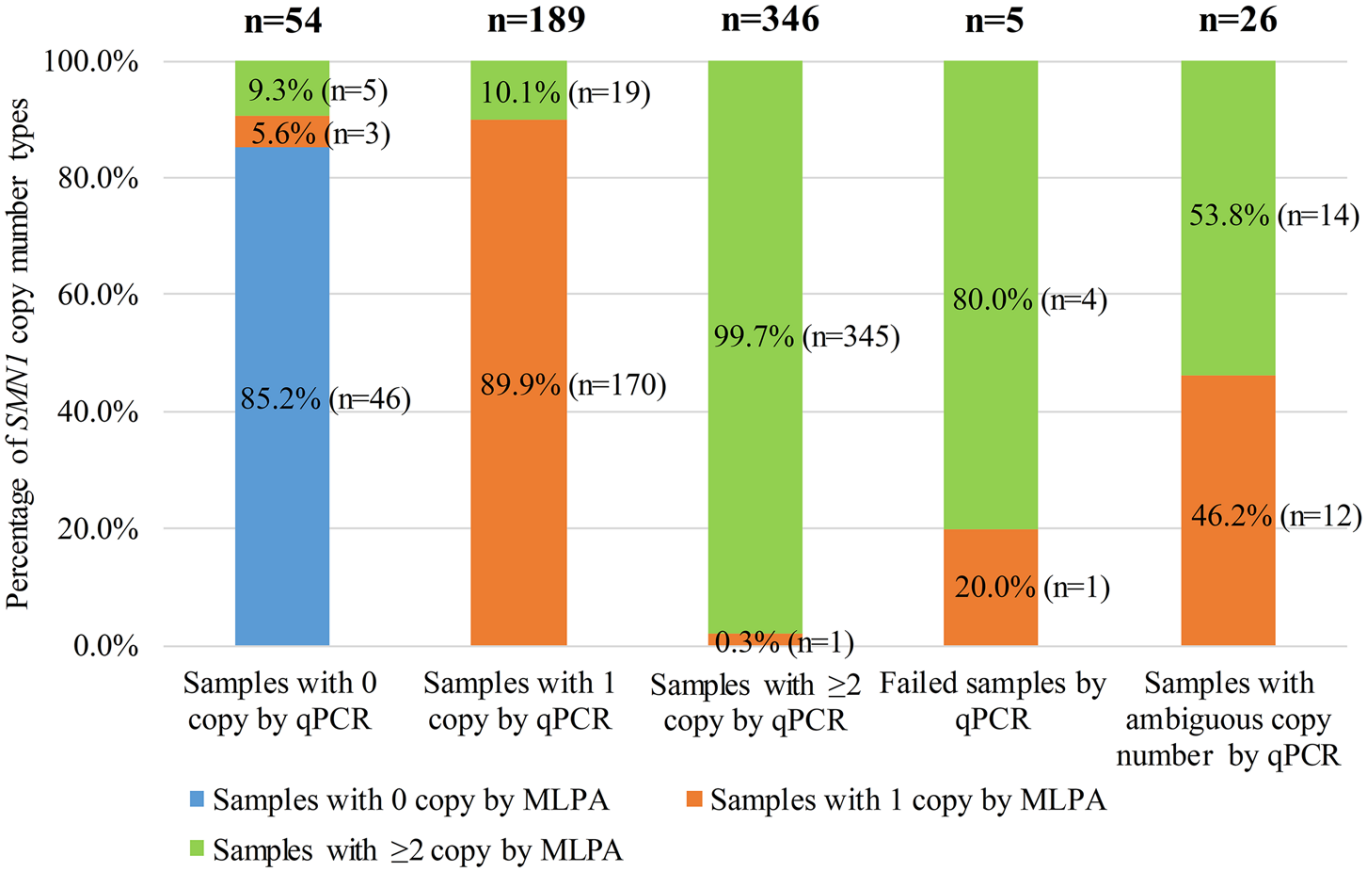
The results of qPCR. A total of 620 reactions were implemented for the 478 samples and 142 repetitions of the 71 repeatability study samples with qPCR. With qPCR method, 26 reactions results were as ambiguous copy numbers, 5 reactions failed, 54 reactions results were 0 copy *SMN1* gene, 189 reactions results were 1 copy *SMN1* gene, and 346 reactions results were ≥ 2 copies *SMN1* gene, each of which is shown as a bar chart. In each bar chart, the samples that were analyzed as 0 copy, 1 copy and ≥ 2 copies of *SMN1* gene with MLPA method are shown as the blue, orange and green, respectively.

**Figure 2:**
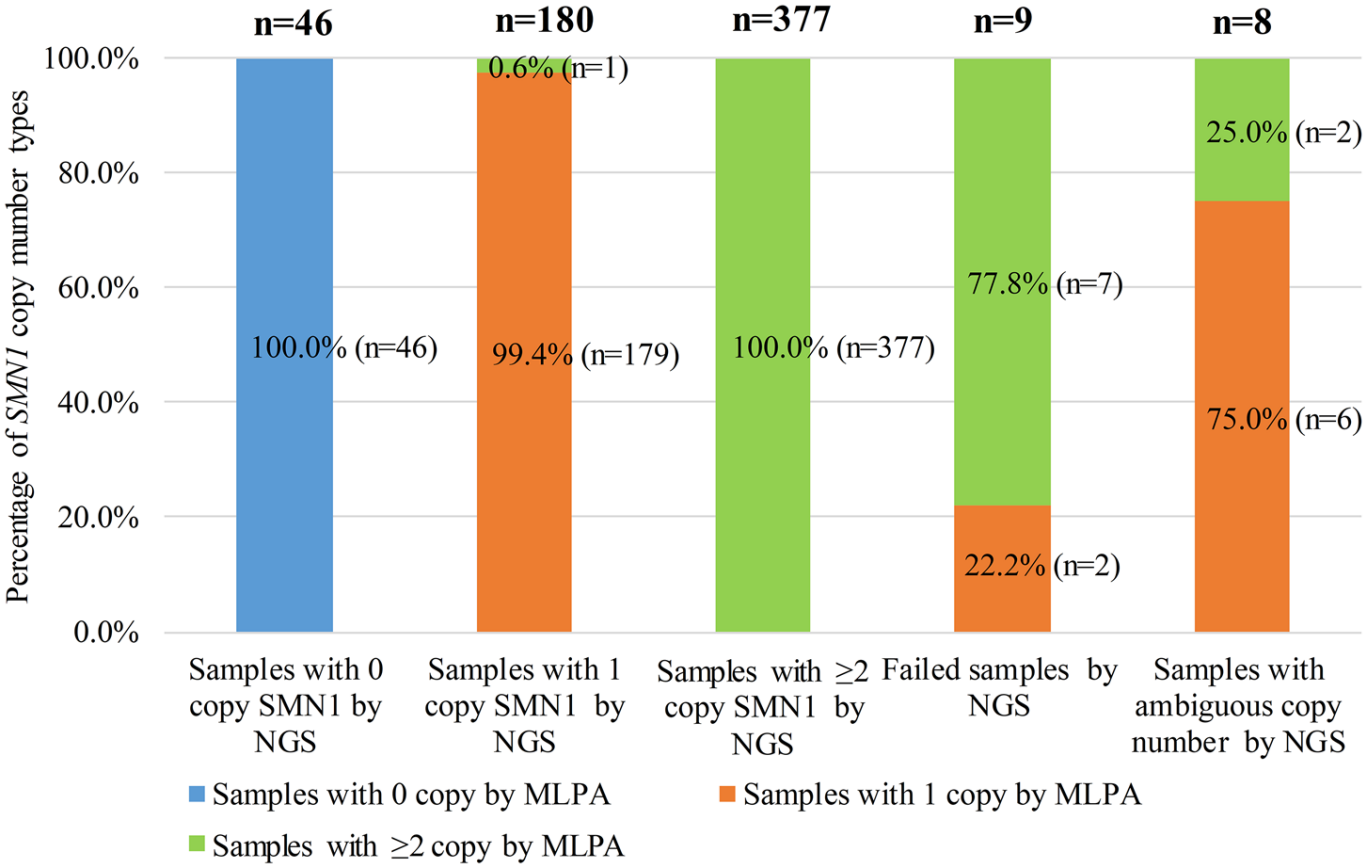
The results of NGS. A total of 620 reactions were implemented for the 478 samples and 142 repetitions of the 71 repeatability study samples with NGS. With NGS method, 8 reactions results were as ambiguous copy numbers, 9 reactions failed, 46 reactions results were 0 copy *SMN1* gene, 180 reactions results were 1 copy *SMN1* gene, and 377 reactions results were ≥ 2 copies *SMN1* gene, each of which is shown as a bar chart. In each bar chart, the samples that were analyzed as 0 copy, 1 copy and ≥ 2 copies of *SMN1* gene with MLPA method are shown as the blue, orange and green, respectively.

Taking the repeatability study experiment into account, for qPCR and NGS methods, a total of 620 reactions were implemented respectively and simultaneously for the 478 samples and 142 repetitions of the 71 repeatability study samples. In the qPCR experiments (Fig. 1), 26 samples were detected as ambiguous copy numbers, and 5 samples failed. In the remaining samples, 54 were classified as homozygous deletion (0 copy of *SMN1*) with qPCR; the results of 8 of these samples were disparate from the MLPA results in which 3 were classified as heterozygous deletion (1 copy) and 5 were classified as normal (≥ 2 copies). A total of 189 samples were classified as heterozygous deletion (1 copy of *SMN1*) by qPCR; the results of 19 of these samples were inconsistent with the MLPA results in which these samples were classified as normal (≥ 2 copies). A total of 346 samples were determined to be nondeletion using qPCR, one of which was determined to be a heterozygous deletion (1 copy) by MLPA. For NGS method (Fig. 2), a total of 9 samples were failed, and 8 samples were analyzed as ambiguous copy numbers, producing 2.74% redetection rate. In the remaining samples, except one false-positive sample in which 2 copies identified by MLPA was detected as one copy by NGS, all the other sample results by NGS were consistent with the MLPA results.

### Repeatability of NGS and qPCR

71 samples (15 of 0 copy, 27 of 1 copy and 29 of ≥ 2 copies of *SMN1*) were analyzed for the reproducibility of the results of the qPCR and NGS methods. Three repeated experiments for each sample were conducted with both qPCR and NGS. The detailed results are presented in Supplement Table S1.

**Figure 3:**
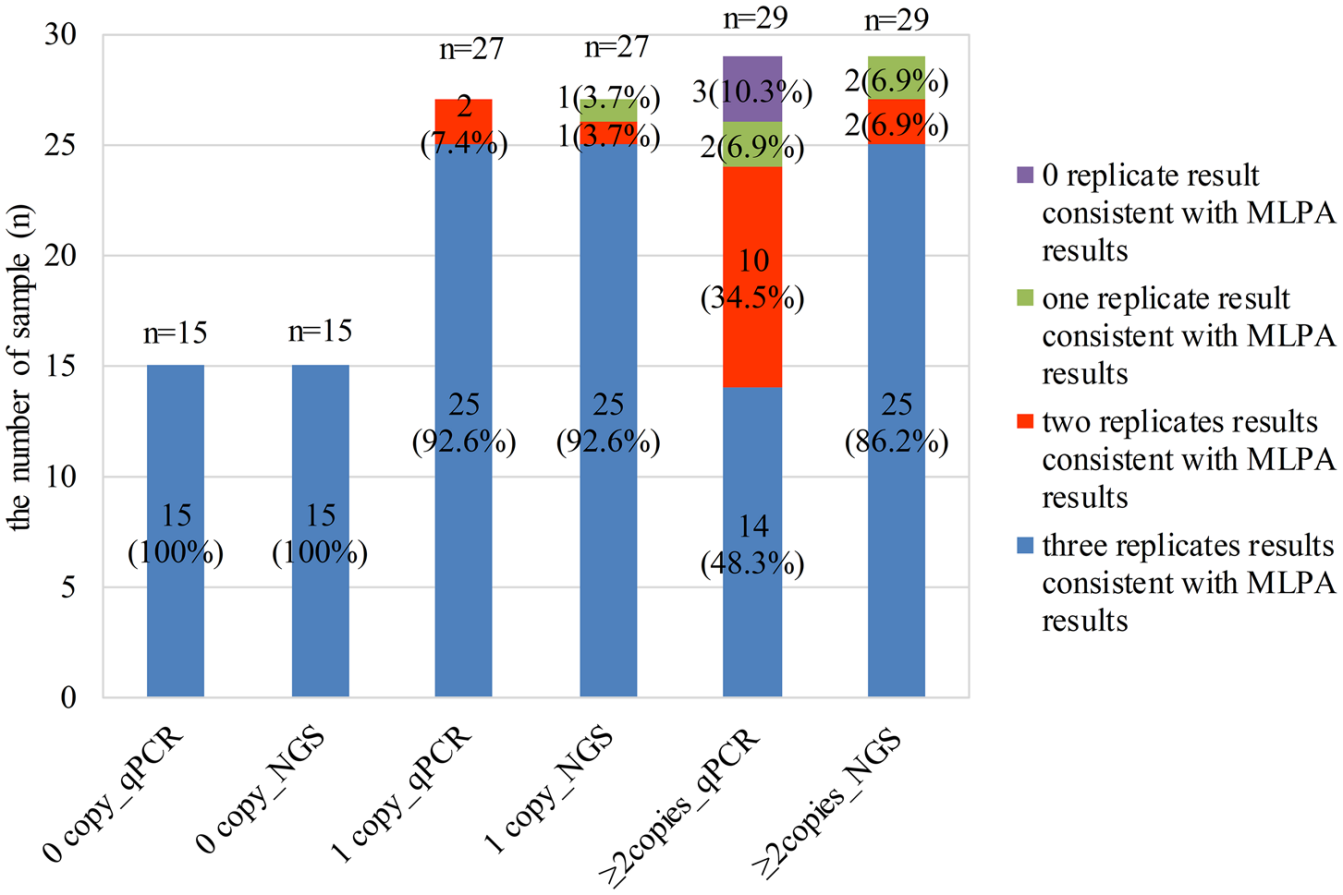
The consistency of repeated experiments compared with the MLPA results. Horizontal axis represents the conditions of 0 copy, 1 copy and ≥ 2 copies of *SMN1* gene detected by qPCR and NGS methods, respectively. Each bar chart represents the sample number used for repeatability study. In each bar chart, blue, red, green, and purple represent samples with three, two, one and 0 replicates result that consistent with MLPA results, respectively.

In the repeated experiment of qPCR, the *SMN1* homozygous deletion was detected in all replicates (Fig. 3), displaying the good reproducibility. For the heterozygous deletion, 92.6% samples were analyzed successfully and accurately in all replicates. In the remaining, one replication was classified as false negative result (Fig. 3). In 29 samples with ≥ 2 copies of *SMN1* gene, only 48.3% samples were tested successfully and accurately in all replicates, and 12 samples were detected accurately in only two replications (34.5%), one replication (6.9%). The remaining (10.3%) were incorrectly detected as deletion in all replications (Fig. 3). For these inconsistent replications, they were detected as heterozygous deletion, homozygous deletion, ambiguous copy numbers or the failed (Supplement Table S1). These results manifested that the reproducibility of nondeletion (≥ 2 copies) samples with qPCR method was unsatisfactory and needed to be further improved.

In the investigation of NGS repeatability, as shown in Fig. 3, the detection rate of homozygous deletion was 100% in all replicates. For heterozygous deletion (1 copy), except one sample with two failed replicates and another sample with one replicate identified as ambiguous copy number, the remaining (92.6%) was accurately analyzed. Among the nondeletion samples (with ≥ 2 copies of *SMN1*), 86.2% were tested successfully and accurately in all replicates. In the remaining 4 samples with 12 replications, three replications were failed, two were ambiguous copy numbers and one was 1 copy (Supplement Table S1). These results suggested that NGS reproducibility performance was significantly superior to that of qPCR.

### Retest rate

In the clinical situation, the samples with the failed and ambiguous results needed to be retested to obtain the definite results. Thus, the retest rate is also an important parameter to evaluate the performance of a methodology. In the first detection run of 478 samples with MLPA, 17 samples were failed detection, and 15 were classified as having ambiguous copy numbers, generating a 6.69% retest rate. For 620 qPCR experiments, 26 samples were detected as ambiguous copy numbers, and 5 samples failed, generating a 5% retest rate. With NGS method, a total of 9 samples failed, and 8 samples had ambiguous copy numbers, producing 2.74% redetection rate, the lowest among the three methods studied. These samples were distributed throughout the collection period (Supplement Table S1), indicating it was not the sample collection time that caused failure or ambiguous results. We only performed retest for the samples with the failed or ambiguous results that were tested using the MLPA method. For 17 failed samples, two of the failures may have been caused by evaporation during the overnight hybridization or ligation step according to the manufacturer’s instructions, and after being retested, the quality control criteria were met. The rest of the samples failed was due to that the standard deviation of the reference probes in the reference samples or the dosage quotient of the reference probes in the test samples did not meet the quality control criteria of the manufacturer’s protocol. After being retested with the previous DNA, 11 samples met the quality control requirements of the protocol, and 4 samples still did not fulfill the quality criteria, while in the second retest with re-extracted DNA, the quality control criteria were met. For 15 samples with ambiguous copy numbers, after the first retest with the previous DNA, 6 of them were still identified as having ambiguous copy numbers, and in the second retest with re-extracted DNA, the results were specific copy numbers of *SMN1* (Supplement Table S1).

### Cost comparison

We compared the reagent cost of three methods. It was about 1.64, 0.61 and 7.23 dollars for MLPA, qPCR and NGS (ECS panel containing more than 200 genes related to 221 genetic diseases). If carrier screening is performed just for only SMA, the reagent cost of qPCR is the lowest, and highest with NGS. Thus, just considering test cost, qPCR is the best option for only SMA one disease carrier screening. However, under the condition of ECS, if cost is divided equally for each disease, the reagent cost of NGS method is the least expensive.

## Discussion

In this study, we comparatively studied three molecular detection methods for *SMN1* copy number. With the same quality template DNA, NGS displayed the lowest retest rate (2.74%), and MLPA and qPCR generated retest rates of 6.69% and 5%, respectively. Moreover, the precision and specificity of NGS were higher than those of qPCR. In addition, in the repeatability study of NGS and qPCR, both showed 100% reproducibility for *SMN1* homozygous deletions, while NGS manifested higher repeatability relative to the qPCR method for the detection of heterozygous deletion and nondeletion of the *SMN1* gene.

The MLPA assay is generally accepted as the gold standard for the molecular analysis of SMA for its high sensitivity and specificity, and it was selected for the validation of the qPCR and NGS methods in this study. It requires DNA free from impurities that may affect MLPA performance due to its greater sensitivity to impurities^26,27^. In most studies of SMA carrier screening, MLPA method was adopted as an independent screening tool^15,28^ or a validated method of positive results detected by other methods^9,29–31^. However, the retest rate of MLPA method has never been researched and analyzed systematically. In this study, the samples failed or with ambiguous results were retested and analyzed. All DNA samples used were extracted and purified with commercial extraction kits (MagPure Buffy Coat DNA Midi KF Kit, MAGEN), which contain magnetic nanoparticles and can effectively remove impurities. For 17 failed samples, after being retested with previous DNA, 13 samples met the quality control requirements of the protocol, illustrating that 13 failed results might be caused by experimental operation problems. With re-extracted DNA, the remaining failed samples fulfilled the quality criteria after being retested, indicating that 4 failed results might be resulted from the poor DNA extraction. For 15 samples with ambiguous copy numbers, according to the retest results, we speculated that 9 of them might be caused by experimental operation problems, and the remaining might be caused by the relatively low DNA quality. Thus, in these retest samples, 22 of them (22/478, 4.6%) might be caused by experimental operation problems, and 10 of them (10/478, 2.09%) might be attributed to the suboptimal DNA quality. These results demonstrated that the MLPA method possessed very strict quality control criteria for high sensitivity and specificity as well as the requirement of high-quality DNA samples. Additionally, for these retest samples (failed or having ambiguous copy numbers by MLPA the first time), 96.88% (31/32) and 71.88% (23/32) of them were successfully and accurately detected by NGS and qPCR method, respectively (Supplement Table S1), which further indicated that the high-quality experimental operation requirements might be the main reason leading to MLPA retest rate. In addition, these results illustrated MLPA method requires higher DNA quality compared with NGS method.

qPCR is a common method used to determine *SMN1* gene copy number^12,14,32–35^. It was selected in this study for a comparative study of performance. Taking repetitive experiments into consideration, a total of 620 qPCR reaction were conducted here in which 5 samples failed and 26 samples were classified as having ambiguous copy numbers, yielding a retest rate of 5%. Thereinto, the ambiguous conditions contributed greatly to the retest rate, accounting for 83.87%. The other qPCR technique^36^ had also been evaluated with 42 samples, and 7 of them (16.6%) were detected as ambiguous copy numbers, while 6 of which were successfully identified as specific copy number (1 or 2 copies) with MLPA (unpublished data). Whether more PCR techniques have the same situation of high proportion of the ambiguous results need further validation and exploration. Up to now, only one study has made systematic statistics on the proportion of the failed and ambiguous results of SMA carrier screening with qPCR method^37^. The failure rate was 3.1%, and 3.3% fell into the equivocal range, most of which was attributed to suboptimal DNA quality and quantity^37^, the retest rate of which (6.4%) is slightly higher than that of this study (5%). In our study, except samples used for repeatability study, the remaining samples failed or with ambiguous results were not be retested. The systematic analysis for the reason of retest would be conducted subsequently. The sensitivity and specificity of PCR-based methods had been reported, ranging from 95.8 to 100% and 83.92 to 100%, respectively^12,23,24,38^. In addition, in a study of newborn screening with qPCR, 8 of 15 tested positive results were confirmed as false-positives with the positive prediction rate of 47%^22^. In our study, for the samples analyzed successfully, 21 tested results were false positives comparing with the MLPA results, generating sensitivity and specificity of 94.30% to 100% and 95.48% to 100%, respectively (Table 1), which is comparative compared with that of the other researches^12,23,24,38^. What’s more, several samples were false positive results in two successive repeated experiment in our study (Supplement Table S1). The differences in specificity between the researches might be related to extraction methods and quality of DNA samples^23^. Furthermore, we speculated that the discrepancy of the specificity and sensitivity between the researches may cause different SMA carrier rates in the same ethnic group^13,14,17,21,35^. Additionally, in the repeatability research, the detection of homozygous deletion was excellent in terms of the repeatability performance of the qPCR method. However, the reproducibility was unsatisfactory for heterozygous deletion and nondeletion samples, especially for nondeletion conditions. For example, three nondeletion samples were detected as heterozygous deletion in three repeated experiments, and another nondeletion sample was identified as heterozygous deletion in the first replication, homozygous deletion in the second replication, and a nondeletion in the third replication. Thus, in general, qPCR method has the advantages of low cost, a short detection period (approximately two days), short process flow and simple operation. Undeniably, not only does this method have a high retest rate but also its accuracy is relatively poor, especially for its reproducibility.

The NGS technique, as a relatively new method, has been developed and gradually applied to the determination of *SMN1* gene copy number, even for SMA carrier screening^18,19,31,39,40^. It displayed good performance not only for the detection of homozygous deletion but also for heterozygous deletion in this study. In 9 failed samples, 8 of them were caused by insufficient sequencing data due to experimental operation problems. After being retested, the remaining one failed sample met the quality control requirements. Moreover, most of the samples that needed to be retested in the MLPA and qPCR tests were successfully tested with the NGS method, illustrating the higher repeatability and the lower requirements of DNA quality for the NGS method. Additionally, the sensitivity, specificity and precision were all 100%, higher than that of qPCR method and in conformity with that of the report^19^. In the repeatability study of 71 samples, for 213 repetitive reactions, 9 (4.23%) repeated experimental results were inconsistent with the MLPA results, 8 of which were classified as failed or as having ambiguous copy numbers, and one was a false-positive result; these inconsistencies were considerably lower than that of the qPCR method (11.74%), indicating higher repeatability of the NGS method compared with the qPCR method. Therefore, the NGS method can be used not only to detect homozygous deletion for SMA genetic diagnosis but also to analyze heterozygous deletion for carrier screening on account of its good performance. Besides, it had been confirmed that NGS method could detect the SNP associated with the “2 + 0” haplotype, accurately determine 3 and 4 copies of *SMN1* gene, as well as detect point mutations of *SMN1* gene^19,20^. What’s more, the copy number of *SMN2* gene could also be analyzed with NGS method^20,41,42^. These detection properties need to be validated with many samples before clinical application. We are collecting and accumulating positive samples for the validation of detection of the SNP associated with the “2 + 0” haplotype, point mutations and ≥ 3 copies of *SMN1* gene.

It is recommended by the ACOG and ACMG that all women and all couples regardless of race or ethnicity undergo carrier screening for SMA, a common severe genetic disease worldwide^6,8^. Thus, the selection of a suitable method is vitally important. Therefore, good performance should be the first priority. In this study, the NGS method showed desirable properties, with the lowest retest rate among the three methods and higher sensitivity, specificity and reproducibility relative to the qPCR method, meeting the performance requirement of SMA carrier screening. Moreover, with NGS technology, ECS has become widely available, accepted and implemented in recent years^17,43,44^. SMA is one of the diseases selected preferentially in the ECS panel. Before the development of *SMN1* copy number analysis with NGS, it has no choice but to supplement additional detection, such as qPCR or MLPA for SMA screening in ECS^13,30,45,46^. This screening mode of combining multiple methods not only increases the screening cost but also raises the complexity of detection compared with a single method, with a negative effect on the reduction of the detection error rate. Thus, the NGS method for *SMN1* copy number analysis is undoubtedly the best option, not only simplifying the process but also reducing the cost relative to the combination of multiple methods.

Except for qPCR, NGS and MLPA, there are other methods that be used for *SMN1* copy number detection. The droplet digital PCR is another emerging technique for *SMN1* copy number assay^47–51^. Although, the improved digital PCR technology has been reported^52^, the most are operational complexity and the related equipment and reagents are expensive. At present, it is mostly used as a validation method for positive results^22,53^ and has not been adopted extensively for carrier screening of SMA. In this study, we performed a comparative study only for three common methods, qPCR, NGS and MLPA, for *SMN1* copy number detection. The NGS method, as a new technique for *SMN1* copy number analysis, displayed desirable performance due to its sensitivity, specificity and repeatability. Moreover, its retest rate was the lowest of the three methods. It was confirmed as a fairly reliable method and would be the most promising method for carrier screening of SMA caused by *SMN1* exon7 deletion. Most importantly, in ECS, the development of the NGS method for *SMN1* copy number analysis could reduce the test cost and simplify the screening process compared with a combination of multiple methods.

## Materials and methods

### DNA samples

A total of 478 DNA samples tested previously with qPCR, MLPA or NGS for exon 7 copy number were enrolled in this study. They were collected from November 2014 to May 2019 from hospitals in mainland China. The informed consent was obtained from all subjects or, if subjects are under 18, from a parent and/or legal guardian. Sixteen samples were from SMA-diagnosed patients with *SMN1* homozygous deletion, 74 samples were from the relatives of SAM patients with *SMN1* heterozygous deletion, and the remaining 388 samples were from individuals without SMA-associated symptoms without family history information. Fifty-nine of the latter 388 samples were previously determined to have heterozygous exon 7 deletion of *SMN1* by qPCR, MLPA or NGS. Genomic DNA was extracted from peripheral whole blood with the MagPure Buffy Coat DNA Midi KF Kit (MAGEN) following the manufacturer’s directions, and subsequently, the extracted DNA was used for the analysis of *SMN1* exon 7 copy number with MLPA, qPCR and NGS simultaneously. This study was approved by the ethics committee of BGI (NO. BGI-IRB 18087).

### NGS and data analysis

A capture array (Integrated DNA Technologies, Inc.) was designed and synthesized to capture the *SMN1* gene and other 237 genes selected for carrier screening. The total size of the targeted region was 1,056,858 bp, and all *SMN1* gene regions were included. For the enrichment of target regions, briefly, genomic DNA was first sheared with fragmentase followed by fragment selection with VAHTS™ DNA Clean Beads (Vazyme, Nanjing, Jiangsu, China). Subsequently, end repair, A-tailing reactions and adapter ligation were implemented. After a post-ligation cleanup, the amplification of fragmented DNA was performed with PCR to harvest sample libraries followed by hybridization with the capture array (Integrated DNA Technologies, Inc.) according to the manufacturer’s protocol. Then, the captured DNA samples were enriched using PCR to obtain the captured libraries, which were sequenced on the MGISEQ-2000 (MGI) platform according to the manufacturer’s instructions.

The primary data were acquired after image processing, error analyses and base calling in the run. Then, unqualified reads and adapter sequences were removed to acquire the clean reads. The alignment of clean reads against the human genome (version hg19/NCBI37 version) was performed with the Burrows Wheeler Aligner software package. The duplicate reads were marked with Picard MarkDuplicates, followed by local realignment and base quality re-correction by GATK. The depth of targeted regions for each sample was generated with GATK DepthOfCoverage.

*SMN1* and *SMN2* copy number analyses were conducted using a previously described protocol^18^ with slight modifications. Briefly, the number of reads that covered several nucleotide difference sites between *SMN1* and *SMN2* was counted, including three *SMN1* loci, namely, chr5: 70,247,773, chr5: 70,247,724 and chr5: 70,247,921, and three *SMN2* loci, namely, chr5: 69,372,353, chr5: 69,372,304 and chr5: 69,372,501. The read number covering exon 7 of the *SMN1* and *SMN2* genes was calculated based on whether the reads contained the *SMN1* base or the *SMN2* base at the difference sites denote as the depth of *SMN1* and *SMN2*. We denote the total depth of *SMN1* and *SMN2* as depth of *SMN*. Before the calculation of the correction coefficient of the exon 7 copy number of *SMN*, we constructed the control sample set that all samples had two copies of exon 7 for both the *SMN1* and *SMN2* genes, and constructed the control gene set that with minimal difference when it’s depth divides into depth of *SMN*. The correction coefficient was calculated for the test samples with 3 steps: firstly, for each sample, we calculated depth ratios that each gene’s depth divides into depth of *SMN*; secondly, for each control gene set, we calculated the mean depth ratio of all control sample set as depth correction coefficient and divided into depth ratio of test sample as gene correction ; finally, we calculate the mean gene correction of all control gene set and get *SMN* exon 7 copy number correction coefficient *θ*, then 4*θ indicate the exon 7 copy number of *SMN*. A Bayesian hierarchical model was used to estimate the exon 7 copy numbers of the *SMN1* and *SMN2* genes. After calculated the confidence interval of the proportion of SMN1 depth p, we use 4*p*θ to estimate the exon 7 copy numbers of the SMN1. For final decision, we use simple thresholds of {0.5, 1.5, 2.5, 3.5} as boundary conditions, while the 95% confidence interval cover one of these thresholds, the result is treated ambiguous.

### MLPA

The MCR-Holland SALSA MLPA Kit P060-050R (MRC-Holland) was used to reidentify the exon 7 copy number according to the manufacturer’s directions. The sequence-specific probes that capture the *SMN1* and *SMN2* genes were included in the kit^54^. The data were analyzed with Coffalyser.NET software (MRC-Holland). Samples with a copy number status were categorized as normal (2 copies), homozygous deletion (0 copies), heterozygous deletion (1 copy), heterozygous duplication (3 copies), homozygous duplication (4 copies) and ambiguous copy number by referring to the manufacturer’s manual.

### qPCR

The *SMN1* exon 7 copy number was analyzed with a TaqMan-MGB qPCR assay kit (Chromysky Medical Research, Shanghai, China) according to the report^30^, which contained specific probes and primers for exon 7 of the *SMN1* gene. qPCR was carried out according to the manufacturer’s recommendations with an ABI StepOne Plus real-time PCR system (Thermo Fisher, USA). The data were analyzed with StepOne Software (v2.3), and the copy number was calculated using the manufacturer’s automated report system (SMASetup_v2.0.2 software, Shanghai Chromysky Medical Research Co., China)-based ΔΔCt method. The results were output as homozygous deletion (0 copies), heterozygous deletion (1 copy), nondeletion (≥ 2 copies), ambiguous copy number and failure.

## Supplementary Information

Supplementary Table S1.

## Data Availability

The data that support the findings of this study are presented in the Supplement Table S1.
