# Supplementary material for: Next generation sequencing is a highly reliable method to analyze exon 7 deletion of survival motor neuron 1 (SMN1) gene
Source: Sci Rep. 2022 Jan 7;12:223. doi: 10.1038/s41598-021-04325-1 (PMC8741787; doi:10.1038/s41598-021-04325-1)
Supplement: Supplementary file 1 — Supplementary Table S1. [file 41598_2021_4325_MOESM1_ESM.pdf]

Supplement Table S1: The copy number of SMN1 gene of 478 samples with MLPA, qPCR and NGS

sample ID	sample collected time (year)	First test for MLPA		Second test for MLPA		Third test for MLPA		qPCR result	NGS result
		QC	copy number of SMN1 gene	QC	copy number of SMN1 gene	QC	copy number of SMN1 gene		
S1	2014	pass	1 copy					0 copy	1 copy
S2	2015	pass	1 copy					1 copy	1 copy
S3	2015	pass	1 copy					1 copy	1 copy
S4	2015	pass	1 copy					1 copy	1 copy
S5	2015	pass	1 copy					1 copy	1 copy
S6	2015	pass	1 copy					1 copy	1 copy
S7	2015	pass	1 copy					1 copy	1 copy
S8	2015	pass	1 copy					1 copy	1 copy
S9	2015	pass	1 copy					1 copy	1 copy
S10	2015	pass	0 copy					0 copy	0 copy
S11	2015	failed		failed		pass	1copy	1 copy	1 copy
S12	2015	pass	1 copy					1 copy	1 copy
S13	2015	pass	1 copy					1 copy	1 copy
S14	2015	pass	1 copy					1 copy	1 copy
S15	2015	pass	1 copy					1 copy	1 copy
S16	2015	pass	1 copy					1 copy	1 copy
S17	2016	pass	1 copy					1 copy	1 copy
S18	2016	pass	1 copy					1 copy	1 copy
S19	2016	pass	1 copy					1 copy	1 copy

S20	2016	pass	ambiguous copy number	pass	1copy			ambiguous copy number	1 copy
S21	2016	pass	ambiguous copy number	ambiguous copy number		pass	1copy	1 copy	1 copy
S22	2016	pass	1 copy					1 copy	1 copy
S23	2016	pass	2 copies					⩾2 copies	⩾2 copies
S24	2016	pass	1 copy					1 copy	1 copy
S25	2016	pass	2 copies					⩾2 copies	⩾2 copies
S26	2016	pass	1 copy					1 copy	1 copy
S27	2016	pass	1 copy					1 copy	1 copy
S28	2016	pass	1 copy					1 copy	1 copy
S29	2016	pass	1 copy					1 copy	1 copy
S30	2016	pass	1 copy					1 copy	1 copy
S31	2016	pass	1 copy					1 copy	1 copy
S32	2016	pass	1 copy					1 copy	1 copy
S33	2016	pass	ambiguous copy number	ambiguous copy number		pass	1copy	1 copy	1 copy
S34	2016	pass	1 copy					1 copy	1 copy
S35	2017	pass	1 copy					1 copy	ambiguous copy number
S36	2017	pass	1 copy					1 copy	1 copy
S37	2017	pass	1 copy					1 copy	1 copy
S38	2017	pass	1 copy					1 copy	1 copy

S39	2017	pass	ambiguous copy number	ambiguous copy number		pass	1copy	1 copy	1 copy
S40	2017	pass	1 copy					1 copy	1 copy
S41	2017	pass	1 copy					1 copy	1 copy
S42	2017	pass	2 copies					⩾2 copies	⩾2 copies
S43	2017	pass	1 copy					1 copy	1 copy
S44	2017	pass	1 copy					1 copy	1 copy
S45	2017	pass	2 copies					⩾2 copies	⩾2 copies
S46	2017	pass	1 copy					ambiguous copy number	1 copy
S47	2017	pass	1 copy					1 copy	1 copy
S48	2017	pass	1 copy					1 copy	1 copy
S49	2017	pass	2 copies					⩾2 copies	⩾2 copies
S50	2017	pass	2 copies					⩾2 copies	⩾2 copies
S51	2017	pass	1 copy					1 copy	1 copy
S52	2017	pass	1 copy					1 copy	1 copy
S53	2017	pass	1 copy					1 copy	1 copy
S54	2017	pass	1 copy					1 copy	1 copy
S55	2017	pass	1 copy					1 copy	1 copy
S56	2017	pass	2 copies					⩾2 copies	⩾2 copies
S57	2017	pass	1 copy					1 copy	1 copy
S58	2017	pass	2 copies					⩾2 copies	⩾2 copies
S59	2017	pass	2 copies					ambiguous copy number	⩾2 copies

S60	2017	pass	1 copy					1 copy	1 copy
S61	2018	pass	2 copies					⩾2 copies	⩾2 copies
S62	2018	pass	2 copies					⩾2 copies	⩾2 copies
S63	2018	pass	2 copies					⩾2 copies	⩾2 copies
S64	2018	pass	2 copies					⩾2 copies	⩾2 copies
S65	2018	pass	2 copies					⩾2 copies	⩾2 copies
S66	2018	pass	3 copies					⩾2 copies	⩾2 copies
S67	2018	pass	2 copies					⩾2 copies	⩾2 copies
S68	2018	pass	1 copy					1 copy	1 copy
S69	2018	pass	1 copy					1 copy	1 copy
S70	2018	pass	2 copies					⩾2 copies	⩾2 copies
S71	2018	failed		pass	2copy			⩾2 copies	⩾2 copies
S72	2018	pass	1 copy					ambiguous copy number	ambiguous copy number
S73	2018	pass	1 copy					ambiguous copy number	1 copy
S74	2018	pass	2 copies					⩾2 copies	⩾2 copies
S75	2018	pass	1 copy					1 copy	1 copy
S76	2018	pass	2 copies					⩾2 copies	⩾2 copies
S77	2018	pass	1 copy					1 copy	1 copy
S78	2018	pass	2 copies					⩾2 copies	⩾2 copies
S79	2018	pass	ambiguous copy number	pass	2copy			1 copy	⩾2 copies
S80	2018	pass	1 copy					1 copy	1 copy
S81	2018	pass	2 copies					⩾2 copies	⩾2 copies

S82	2018	pass	2 copies					⩾2 copies	⩾2 copies
S83	2018	pass	2 copies					⩾2 copies	⩾2 copies
S84	2018	pass	2 copies					⩾2 copies	⩾2 copies
S85	2018	pass	2 copies					⩾2 copies	⩾2 copies
S86	2018	pass	2 copies					⩾2 copies	⩾2 copies
S87	2018	pass	2 copies					⩾2 copies	⩾2 copies
S88	2018	pass	2 copies					⩾2 copies	⩾2 copies
S89	2018	pass	3 copies					⩾2 copies	⩾2 copies
S90	2018	pass	1 copy					1 copy	1 copy
S91	2018	pass	2 copies					⩾2 copies	⩾2 copies
S92	2018	pass	2 copies					⩾2 copies	⩾2 copies
S93	2018	pass	2 copies					⩾2 copies	⩾2 copies
S94	2018	pass	1 copy					1 copy	1 copy
S95	2018	pass	2 copies					⩾2 copies	⩾2 copies
S96	2018	pass	2 copies					⩾2 copies	⩾2 copies
S97	2018	pass	2 copies					ambiguous copy number	⩾2 copies
S98	2018	failed		pass	2copy			⩾2 copies	⩾2 copies
S99	2018	pass	2 copies					⩾2 copies	⩾2 copies
S100	2018	pass	2 copies					⩾2 copies	⩾2 copies
S101	2018	pass	3 copies					⩾2 copies	⩾2 copies
S102	2018	pass	2 copies					⩾2 copies	⩾2 copies
S103	2018	pass	2 copies					⩾2 copies	⩾2 copies
S104	2018	pass	2 copies					⩾2 copies	⩾2 copies
S105	2018	pass	3 copies					⩾2 copies	⩾2 copies

S106	2018	pass	2 copies					⩾2 copies	⩾2 copies
S107	2018	pass	2 copies					⩾2 copies	⩾2 copies
S108	2018	pass	2 copies					⩾2 copies	⩾2 copies
S109	2018	pass	2 copies					⩾2 copies	failed
S110	2018	pass	2 copies					⩾2 copies	failed
S111	2018	pass	1 copy					ambiguous copy number	1 copy
S112	2018	pass	2 copies					⩾2 copies	⩾2 copies
S113	2018	pass	3 copies					⩾2 copies	⩾2 copies
S114	2018	pass	2 copies					⩾2 copies	⩾2 copies
S115	2018	pass	3 copies					⩾2 copies	⩾2 copies
S116	2018	pass	2 copies					⩾2 copies	⩾2 copies
S117	2018	pass	2 copies					⩾2 copies	⩾2 copies
S118	2018	pass	2 copies					⩾2 copies	⩾2 copies
S119	2018	pass	2 copies					⩾2 copies	⩾2 copies
S120	2018	pass	2 copies					failed	⩾2 copies
S121	2018	pass	2 copies					⩾2 copies	⩾2 copies
S122	2018	pass	2 copies					⩾2 copies	⩾2 copies
S123	2018	pass	3 copies					⩾2 copies	failed
S124	2018	pass	2 copies					⩾2 copies	⩾2 copies
S125	2018	pass	2 copies					⩾2 copies	⩾2 copies
S126	2018	pass	2 copies					⩾2 copies	⩾2 copies
S127	2018	pass	2 copies					⩾2 copies	⩾2 copies
S128	2018	pass	2 copies					⩾2 copies	⩾2 copies
S129	2018	pass	2 copies					⩾2 copies	⩾2 copies

S130	2018	pass	2 copies					⩾2 copies	⩾2 copies
S131	2018	pass	2 copies					⩾2 copies	⩾2 copies
S132	2018	pass	2 copies					⩾2 copies	⩾2 copies
S133	2018	pass	2 copies					⩾2 copies	⩾2 copies
S134	2018	pass	ambiguous copy number	ambiguous copy number		pass	2copy	ambiguous copy number	⩾2 copies
S135	2018	pass	2 copies					⩾2 copies	failed
S136	2018	pass	2 copies					⩾2 copies	⩾2 copies
S137	2018	pass	2 copies					⩾2 copies	⩾2 copies
S138	2018	pass	2 copies					⩾2 copies	⩾2 copies
S139	2018	pass	2 copies					⩾2 copies	⩾2 copies
S140	2018	pass	2 copies					⩾2 copies	⩾2 copies
S141	2018	pass	1 copy					1 copy	1 copy
S142	2018	pass	1 copy					0 copy	1 copy
S143	2018	pass	1 copy					1 copy	1 copy
S144	2018	pass	2 copies					ambiguous copy number	⩾2 copies
S145	2018	pass	2 copies					⩾2 copies	⩾2 copies
S146	2018	pass	1 copy					1 copy	1 copy
S147	2018	pass	2 copies					⩾2 copies	⩾2 copies
S148	2018	pass	1 copy					1 copy	1 copy
S149	2018	pass	2 copies					⩾2 copies	⩾2 copies
S150	2018	pass	2 copies					⩾2 copies	⩾2 copies
S151	2018	pass	1 copy					1 copy	1 copy

S152	2018	pass	2 copies					⩾2 copies	⩾2 copies
S153	2018	pass	3 copies					⩾2 copies	⩾2 copies
S154	2018	pass	2 copies					⩾2 copies	⩾2 copies
S155	2018	pass	2 copies					⩾2 copies	⩾2 copies
S156	2018	pass	1 copy					1 copy	1 copy
S157	2018	pass	2 copies					⩾2 copies	⩾2 copies
S158	2018	pass	2 copies					⩾2 copies	⩾2 copies
S159	2018	pass	1 copy					1 copy	1 copy
S160	2018	pass	1 copy					1 copy	1 copy
S161	2018	pass	1 copy					1 copy	1 copy
S162	2018	pass	2 copies					⩾2 copies	⩾2 copies
S163	2018	pass	2 copies					⩾2 copies	⩾2 copies
S164	2018	pass	1 copy					1 copy	1 copy
S165	2018	pass	2 copies					ambiguous copy number	⩾2 copies
S166	2018	pass	2 copies					⩾2 copies	⩾2 copies
S167	2018	pass	1 copy					1 copy	1 copy
S168	2018	pass	ambiguous copy number	ambiguous copy number		pass	1copy	1 copy	1 copy
S169	2018	pass	1 copy					1 copy	1 copy
S170	2018	pass	1 copy					1 copy	1 copy
S171	2018	pass	2 copies					ambiguous copy number	⩾2 copies
S172	2018	pass	1 copy					1 copy	1 copy

S173	2018	pass	2 copies					⩾2 copies	⩾2 copies
S174	2018	pass	2 copies					⩾2 copies	⩾2 copies
S175	2018	pass	2 copies					⩾2 copies	⩾2 copies
S176	2018	pass	1 copy					1 copy	1 copy
S177	2018	pass	1 copy					1 copy	1 copy
S178	2018	pass	2 copies					⩾2 copies	⩾2 copies
S179	2018	pass	1 copy					1 copy	1 copy
S180	2018	pass	2 copies					⩾2 copies	⩾2 copies
S181	2018	pass	1 copy					1 copy	1 copy
S182	2018	pass	2 copies					⩾2 copies	⩾2 copies
S183	2018	pass	2 copies					⩾2 copies	⩾2 copies
S184	2018	pass	2 copies					⩾2 copies	⩾2 copies
S185	2018	pass	1 copy					1 copy	1 copy
S186	2018	pass	1 copy					1 copy	1 copy
S187	2018	pass	2 copies					⩾2 copies	⩾2 copies
S188	2018	pass	2 copies					⩾2 copies	⩾2 copies
S189	2018	pass	1 copy					ambiguous copy number	1 copy
S190	2018	pass	2 copies					⩾2 copies	⩾2 copies
S191	2018	pass	2 copies					⩾2 copies	⩾2 copies
S192	2018	pass	1 copy					1 copy	1 copy
S193	2018	pass	1 copy					1 copy	1 copy
S194	2018	pass	2 copies					⩾2 copies	⩾2 copies
S195	2018	pass	1 copy					1 copy	1 copy
S196	2018	pass	1 copy					1 copy	1 copy

S197	2018	pass	1 copy					1 copy	1 copy
S198	2018	pass	2 copies					⩾2 copies	⩾2 copies
S199	2018	pass	1 copy					ambiguous copy number	1 copy
S200	2018	pass	2 copies					⩾2 copies	⩾2 copies
S201	2018	pass	2 copies					⩾2 copies	⩾2 copies
S202	2018	pass	2 copies					⩾2 copies	⩾2 copies
S203	2018	pass	2 copies					⩾2 copies	⩾2 copies
S204	2018	pass	2 copies					1 copy	⩾2 copies
S205	2018	pass	1 copy					1 copy	1 copy
S206	2018	pass	2 copies					⩾2 copies	⩾2 copies
S207	2018	pass	2 copies					⩾2 copies	⩾2 copies
S208	2018	pass	2 copies					⩾2 copies	⩾2 copies
S209	2018	pass	2 copies					⩾2 copies	⩾2 copies
S210	2018	pass	2 copies					⩾2 copies	⩾2 copies
S211	2018	pass	2 copies					⩾2 copies	⩾2 copies
S212	2018	pass	2 copies					⩾2 copies	⩾2 copies
S213	2018	pass	2 copies					⩾2 copies	⩾2 copies
S214	2018	pass	2 copies					⩾2 copies	⩾2 copies
S215	2018	pass	3 copies					⩾2 copies	⩾2 copies
S216	2018	pass	3 copies					⩾2 copies	⩾2 copies
S217	2018	pass	2 copies					⩾2 copies	⩾2 copies
S218	2018	pass	2 copies					⩾2 copies	⩾2 copies
S219	2018	pass	2 copies					⩾2 copies	⩾2 copies
S220	2018	pass	3 copies					⩾2 copies	⩾2 copies

S221	2018	pass	2 copies					ambiguous copy number	⩾2 copies
S222	2018	pass	2 copies					⩾2 copies	⩾2 copies
S223	2018	pass	2 copies					⩾2 copies	⩾2 copies
S224	2018	pass	2 copies					⩾2 copies	⩾2 copies
S225	2018	failed		pass	2copy			⩾2 copies	⩾2 copies
S226	2018	pass	2 copies					⩾2 copies	⩾2 copies
S227	2018	pass	2 copies					⩾2 copies	⩾2 copies
S228	2018	pass	2 copies					⩾2 copies	⩾2 copies
S229	2018	pass	2 copies					⩾2 copies	⩾2 copies
S230	2018	pass	ambiguous copy number	pass	2copy			1 copy	⩾2 copies
S231	2018	pass	3 copies					⩾2 copies	⩾2 copies
S232	2018	pass	2 copies					⩾2 copies	⩾2 copies
S233	2018	pass	2 copies					⩾2 copies	⩾2 copies
S234	2018	pass	2 copies					⩾2 copies	⩾2 copies
S235	2018	pass	2 copies					⩾2 copies	⩾2 copies
S236	2018	pass	2 copies					⩾2 copies	⩾2 copies
S237	2018	pass	2 copies					⩾2 copies	⩾2 copies
S238	2018	pass	2 copies					⩾2 copies	⩾2 copies
S239	2018	pass	2 copies					⩾2 copies	⩾2 copies
S240	2018	pass	3 copies					⩾2 copies	⩾2 copies
S241	2018	pass	1 copy					1 copy	1 copy
S242	2018	pass	1 copy					0 copy	ambiguous copy number

S243	2018	pass	2 copies					⩾2 copies	⩾2 copies
S244	2018	pass	2 copies					⩾2 copies	⩾2 copies
S245	2018	pass	2 copies					⩾2 copies	⩾2 copies
S246	2018	pass	2 copies					⩾2 copies	⩾2 copies
S247	2018	pass	2 copies					⩾2 copies	⩾2 copies
S248	2018	pass	2 copies					⩾2 copies	⩾2 copies
S249	2018	pass	2 copies					⩾2 copies	⩾2 copies
S250	2018	pass	2 copies					⩾2 copies	⩾2 copies
S251	2018	pass	2 copies					⩾2 copies	⩾2 copies
S252	2018	pass	2 copies					⩾2 copies	⩾2 copies
S253	2018	pass	2 copies					ambiguous copy number	⩾2 copies
S254	2018	pass	2 copies					⩾2 copies	⩾2 copies
S255	2018	pass	2 copies					⩾2 copies	⩾2 copies
S256	2018	pass	2 copies					⩾2 copies	⩾2 copies
S257	2018	pass	1 copy					failed	1 copy
S258	2018	pass	2 copies					⩾2 copies	⩾2 copies
S259	2018	pass	2 copies					⩾2 copies	⩾2 copies
S260	2018	pass	2 copies					⩾2 copies	⩾2 copies
S261	2018	pass	2 copies					⩾2 copies	⩾2 copies
S262	2018	pass	2 copies					⩾2 copies	⩾2 copies
S263	2018	pass	2 copies					⩾2 copies	⩾2 copies
S264	2018	pass	2 copies					⩾2 copies	⩾2 copies
S265	2018	pass	2 copies					⩾2 copies	⩾2 copies
S266	2018	pass	2 copies					⩾2 copies	⩾2 copies

S267	2018	pass	2 copies					⩾2 copies	⩾2 copies
S268	2018	pass	2 copies					⩾2 copies	⩾2 copies
S269	2018	pass	3 copies					⩾2 copies	⩾2 copies
S270	2018	pass	2 copies					⩾2 copies	⩾2 copies
S271	2018	pass	1 copy					1 copy	1 copy
S272	2018	pass	2 copies					⩾2 copies	⩾2 copies
S273	2018	pass	2 copies					⩾2 copies	⩾2 copies
S274	2018	pass	2 copies					ambiguous copy number	⩾2 copies
S275	2018	pass	2 copies					⩾2 copies	⩾2 copies
S276	2018	pass	2 copies					⩾2 copies	⩾2 copies
S277	2018	pass	2 copies					⩾2 copies	⩾2 copies
S278	2018	pass	2 copies					⩾2 copies	⩾2 copies
S279	2018	pass	2 copies					⩾2 copies	⩾2 copies
S280	2018	pass	2 copies					⩾2 copies	⩾2 copies
S281	2018	pass	3 copies					⩾2 copies	⩾2 copies
S282	2018	pass	2 copies					⩾2 copies	⩾2 copies
S283	2018	pass	2 copies					⩾2 copies	⩾2 copies
S284	2018	pass	2 copies					⩾2 copies	⩾2 copies
S285	2018	pass	2 copies					⩾2 copies	⩾2 copies
S286	2018	pass	2 copies					⩾2 copies	⩾2 copies
S287	2018	pass	2 copies					⩾2 copies	⩾2 copies
S288	2018	pass	2 copies					⩾2 copies	⩾2 copies
S289	2018	pass	2 copies					⩾2 copies	⩾2 copies
S290	2018	pass	3 copies					⩾2 copies	⩾2 copies

S291	2018	pass	2 copies					⩾2 copies	⩾2 copies
S292	2018	pass	2 copies					⩾2 copies	⩾2 copies
S293	2018	pass	2 copies					⩾2 copies	⩾2 copies
S294	2018	pass	2 copies					⩾2 copies	⩾2 copies
S295	2018	pass	1 copy					ambiguous copy number	1 copy
S296	2018	pass	1 copy					ambiguous copy number	ambiguous copy number
S297	2018	pass	2 copies					⩾2 copies	⩾2 copies
S298	2018	pass	2 copies					⩾2 copies	⩾2 copies
S299	2018	pass	2 copies					⩾2 copies	⩾2 copies
S300	2018	pass	2 copies					⩾2 copies	⩾2 copies
S301	2018	pass	2 copies					⩾2 copies	⩾2 copies
S302	2018	pass	2 copies					⩾2 copies	⩾2 copies
S303	2018	pass	2 copies					⩾2 copies	⩾2 copies
S304	2018	pass	2 copies					⩾2 copies	⩾2 copies
S305	2018	pass	2 copies					⩾2 copies	⩾2 copies
S306	2018	pass	2 copies					⩾2 copies	⩾2 copies
S307	2019	pass	1 copy					1 copy	1 copy
S308	2019	pass	1 copy					1 copy	1 copy
S309	2019	pass	1 copy					ambiguous copy number	1 copy
S310	2019	pass	2 copies					⩾2 copies	⩾2 copies
S311	2019	pass	2 copies					⩾2 copies	⩾2 copies
S312	2019	pass	2 copies					⩾2 copies	⩾2 copies

S313	2019	pass	2 copies					≥2 copies	≥2 copies
S314	2019	pass	1 copy					1 copy	ambiguous copy number
S315	2019	pass	1 copy					1 copy	1 copy
S316	2019	pass	2 copies					≥2 copies	≥2 copies
S317	2019	pass	2 copies					≥2 copies	≥2 copies
S318	2019	pass	2 copies					≥2 copies	≥2 copies
S319	2019	failed		failed		pass	2copy	≥2 copies	≥2 copies
S320	2019	pass	2 copies					≥2 copies	≥2 copies
S321	2019	pass	1 copy					ambiguous copy number	1 copy
S322	2019	pass	1 copy					1 copy	1 copy
S323	2019	pass	2 copies					≥2 copies	≥2 copies
S324	2019	pass	2 copies					≥2 copies	≥2 copies
S325	2019	pass	2 copies					≥2 copies	≥2 copies
S326	2019	pass	1 copy					1 copy	1 copy
S327	2019	pass	2 copies					≥2 copies	≥2 copies
S328	2019	pass	2 copies					≥2 copies	≥2 copies
S329	2019	pass	2 copies					≥2 copies	≥2 copies
S330	2019	pass	2 copies					0 copy	≥2 copies
S331	2019	pass	3 copies					≥2 copies	≥2 copies
S332	2019	pass	2 copies					≥2 copies	≥2 copies
S333	2019	pass	2 copies					failed	≥2 copies
S334	2019	pass	2 copies					≥2 copies	≥2 copies
S335	2019	pass	2 copies					≥2 copies	≥2 copies

S336	2019	pass	2 copies					⩾2 copies	⩾2 copies
S337	2019	pass	2 copies					⩾2 copies	⩾2 copies
S338	2019	pass	1 copy					1 copy	1 copy
S339	2019	pass	1 copy					1 copy	1 copy
S340	2019	pass	2 copies					⩾2 copies	⩾2 copies
S341	2019	pass	2 copies					⩾2 copies	⩾2 copies
S342	2019	pass	2 copies					⩾2 copies	⩾2 copies
S343	2019	pass	2 copies					⩾2 copies	⩾2 copies
S344	2019	pass	2 copies					⩾2 copies	⩾2 copies
S345	2019	pass	2 copies					⩾2 copies	⩾2 copies
S346	2019	pass	2 copies					⩾2 copies	⩾2 copies
S347	2019	pass	2 copies					⩾2 copies	⩾2 copies
S348	2019	pass	2 copies					⩾2 copies	⩾2 copies
S349	2019	pass	2 copies					⩾2 copies	⩾2 copies
S350	2019	pass	2 copies					⩾2 copies	⩾2 copies
S351	2019	pass	2 copies					⩾2 copies	⩾2 copies
S352	2019	pass	2 copies					⩾2 copies	⩾2 copies
S353	2019	pass	2 copies					⩾2 copies	⩾2 copies
S354	2019	pass	3 copies					⩾2 copies	⩾2 copies
S355	2019	pass	2 copies					⩾2 copies	⩾2 copies
S356	2019	failed		pass	3copy			⩾2 copies	⩾2 copies
S357	2019	pass	2 copies					⩾2 copies	⩾2 copies
S358	2019	pass	2 copies					⩾2 copies	⩾2 copies
S359	2019	pass	2 copies					⩾2 copies	⩾2 copies
S360	2019	pass	2 copies					⩾2 copies	⩾2 copies

S361	2019	pass	ambiguous copy number	pass	2copy			⩾2 copies	⩾2 copies
S362	2019	pass	2 copies					⩾2 copies	⩾2 copies
S363	2019	failed		pass	3copy			0 copy	⩾2 copies
S364	2019	pass	2 copies					⩾2 copies	⩾2 copies
S365	2019	failed		pass	2copy			⩾2 copies	⩾2 copies
S366	2019	pass	ambiguous copy number	pass	2copy			⩾2 copies	⩾2 copies
S367	2019	failed		pass	2copy			⩾2 copies	⩾2 copies
S368	2019	pass	ambiguous copy number	pass	2copy			⩾2 copies	⩾2 copies
S369	2019	failed		pass	3copy			⩾2 copies	⩾2 copies
S370	2019	failed		failed		pass	3copy	0 copy	⩾2 copies
S371	2019	failed		pass	2copy			⩾2 copies	⩾2 copies
S372	2019	pass	2 copies					failed	⩾2 copies
S373	2019	pass	2 copies					failed	⩾2 copies
S374	2019	pass	2 copies					⩾2 copies	⩾2 copies
S375	2019	pass	2 copies					⩾2 copies	⩾2 copies
S376	2019	pass	2 copies					⩾2 copies	⩾2 copies
S377	2019	pass	2 copies					⩾2 copies	⩾2 copies
S378	2019	pass	2 copies					⩾2 copies	⩾2 copies
S379	2019	pass	2 copies					⩾2 copies	⩾2 copies
S380	2019	pass	2 copies					⩾2 copies	⩾2 copies
S381	2019	pass	2 copies					⩾2 copies	⩾2 copies
S382	2019	pass	2 copies					⩾2 copies	⩾2 copies

S383	2019	pass	2 copies					⩾2 copies	⩾2 copies
S384	2019	pass	2 copies					⩾2 copies	⩾2 copies
S385	2019	pass	2 copies					⩾2 copies	⩾2 copies
S386	2019	pass	2 copies					⩾2 copies	⩾2 copies
S387	2019	pass	3 copies					⩾2 copies	⩾2 copies
S388	2019	pass	2 copies					⩾2 copies	⩾2 copies
S389	2019	failed		pass	2copy			⩾2 copies	⩾2 copies
S390	2019	pass	2 copies					⩾2 copies	⩾2 copies
S391	2019	pass	2 copies					⩾2 copies	⩾2 copies
S392	2019	failed		pass	2copy			⩾2 copies	⩾2 copies
S393	2019	pass	2 copies					⩾2 copies	⩾2 copies
S394	2019	failed		failed		pass	2copy	⩾2 copies	⩾2 copies
S395	2019	pass	2 copies					⩾2 copies	⩾2 copies
S396	2019	pass	2 copies					⩾2 copies	⩾2 copies
S397	2019	pass	2 copies					⩾2 copies	⩾2 copies
S398	2019	failed		pass	2copy			⩾2 copies	⩾2 copies
S399	2019	pass	3 copies					⩾2 copies	⩾2 copies
S400	2019	failed		pass	2copy			⩾2 copies	⩾2 copies
S401	2019	pass	2 copies					⩾2 copies	⩾2 copies
S402	2019	pass	2 copies					⩾2 copies	⩾2 copies
S403	2019	pass	2 copies					⩾2 copies	⩾2 copies
S404	2019	pass	2 copies					⩾2 copies	⩾2 copies
S405	2019	pass	2 copies					⩾2 copies	⩾2 copies
S406	2019	pass	2 copies					⩾2 copies	⩾2 copies
S407	2019	pass	2 copies					⩾2 copies	⩾2 copies

S408_1	2015	pass	1 copy					1 copy	1 copy
S408_2	2015							1 copy	1 copy
S408_3	2015							1 copy	1 copy
S409_1	2015	pass	1 copy					1 copy	1 copy
S409_2	2015							1 copy	1 copy
S409_3	2015							1 copy	1 copy
S410_1	2015	pass	1 copy					1 copy	1 copy
S410_2	2015							1 copy	1 copy
S410_3	2015							1 copy	1 copy
S411_1	2015	pass	1 copy					1 copy	1 copy
S411_2	2015							1 copy	1 copy
S411_3	2015							1 copy	1 copy
S412_1	2016	pass	0 copy					0 copy	0 copy
S412_2	2016							0 copy	0 copy
S412_3	2016							0 copy	0 copy
S413_1	2016	pass	0 copy					0 copy	0 copy
S413_2	2016							0 copy	0 copy
S413_3	2016							0 copy	0 copy
S414_1	2016	pass	0 copy					0 copy	0 copy
S414_2	2016							0 copy	0 copy
S414_3	2016							0 copy	0 copy
S415_1	2016	pass	0 copy					0 copy	0 copy
S415_2	2016							0 copy	0 copy
S415_3	2016							0 copy	0 copy
S416_1	2016	pass	0 copy					0 copy	0 copy

S416_2	2016							0 copy	0 copy
S416_3	2016							0 copy	0 copy
S417_1	2016	pass	1 copy					1 copy	1 copy
S417_2	2016							1 copy	1 copy
S417_3	2016							1 copy	1 copy
S418_1	2016	pass	1 copy					1 copy	1 copy
S418_2	2016							1 copy	1 copy
S418_3	2016							1 copy	1 copy
S419_1	2017	pass	0 copy					0 copy	0 copy
S419_2	2017							0 copy	0 copy
S419_3	2017							0 copy	0 copy
S420_1	2017	pass	0 copy					0 copy	0 copy
S420_2	2017							0 copy	0 copy
S420_3	2017							0 copy	0 copy
S421_1	2017	pass	0 copy					0 copy	0 copy
S421_2	2017							0 copy	0 copy
S421_3	2017							0 copy	0 copy
S422_1	2017	pass	0 copy					0 copy	0 copy
S422_2	2017							0 copy	0 copy
S422_3	2017							0 copy	0 copy
S423_1	2018	pass	2 copies					1 copy	⩾2 copies
S423_2	2018							1 copy	⩾2 copies
S423_3	2018							⩾2 copies	⩾2 copies
S424_1	2018	pass	0 copy					0 copy	0 copy
S424_2	2018							0 copy	0 copy

S424_3	2018							0 copy	0 copy
S425_1	2018	pass	2 copies					1 copy	⩾2 copies
S425_2	2018							ambiguous copy number	⩾2 copies
S425_3	2018							ambiguous copy number	⩾2 copies
S426_1	2018	pass	2 copies					1 copy	⩾2 copies
S426_2	2018							⩾2 copies	⩾2 copies
S426_3	2018							⩾2 copies	⩾2 copies
S427_1	2018	pass	0 copy					0 copy	0 copy
S427_2	2018							0 copy	0 copy
S427_3	2018							0 copy	0 copy
S428_1	2018	pass	2 copies					⩾2 copies	⩾2 copies
S428_2	2018							⩾2 copies	⩾2 copies
S428_3	2018							⩾2 copies	⩾2 copies
S429_1	2018	pass	2 copies					1 copy	⩾2 copies
S429_2	2018							⩾2 copies	⩾2 copies
S429_3	2018							⩾2 copies	⩾2 copies
S430_1	2018	pass	2 copies					⩾2 copies	ambiguous copy number
S430_2	2018							⩾2 copies	⩾2 copies
S430_3	2018							⩾2 copies	⩾2 copies
S431_1	2018	pass	3 copies					⩾2 copies	⩾2 copies
S431_2	2018							⩾2 copies	⩾2 copies
S431_3	2018							⩾2 copies	⩾2 copies

S432_1	2018	pass	2 copies					⩾2 copies	⩾2 copies
S432_2	2018							⩾2 copies	⩾2 copies
S432_3	2018							⩾2 copies	⩾2 copies
S433_1	2018	pass	2 copies					⩾2 copies	⩾2 copies
S433_2	2018							⩾2 copies	⩾2 copies
S433_3	2018							⩾2 copies	⩾2 copies
S434_1	2018	pass	2 copies					⩾2 copies	⩾2 copies
S434_2	2018							⩾2 copies	⩾2 copies
S434_3	2018							⩾2 copies	⩾2 copies
S435_1	2018	pass	2 copies					⩾2 copies	⩾2 copies
S435_2	2018							1 copy	⩾2 copies
S435_3	2018							⩾2 copies	⩾2 copies
S436_1	2018	pass	2 copies					⩾2 copies	failed
S436_2	2018							⩾2 copies	⩾2 copies
S436_3	2018							⩾2 copies	failed
S437_1	2018	pass	2 copies					⩾2 copies	⩾2 copies
S437_2	2018							0 copy	⩾2 copies
S437_3	2018							⩾2 copies	⩾2 copies
S438_1	2018	pass	ambiguous copy number	ambiguous copy number		pass	2copy	1 copy	failed
S438_2	2018							1 copy	⩾2 copies
S438_3	2018							1 copy	⩾2 copies
S439_1	2018	pass	ambiguous copy number	pass	2copy			1 copy	⩾2 copies

S439_2	2018							ambiguous copy number	⩾2 copies
S439_3	2018							ambiguous copy number	⩾2 copies
S440_1	2018	pass	2 copies					⩾2 copies	⩾2 copies
S440_2	2018							⩾2 copies	⩾2 copies
S440_3	2018							⩾2 copies	⩾2 copies
S441_1	2018	pass	2 copies					⩾2 copies	⩾2 copies
S441_2	2018							⩾2 copies	⩾2 copies
S441_3	2018							⩾2 copies	⩾2 copies
S442_1	2018	pass	0 copy					0 copy	0 copy
S442_2	2018							0 copy	0 copy
S442_3	2018							0 copy	0 copy
S443_1	2018	pass	2 copies					1 copy	⩾2 copies
S443_2	2018							0 copy	⩾2 copies
S443_3	2018							⩾2 copies	⩾2 copies
S444_1	2018	pass	2 copies					1 copy	⩾2 copies
S444_2	2018							⩾2 copies	⩾2 copies
S444_3	2018							⩾2 copies	⩾2 copies
S445_1	2018	pass	1 copy					1 copy	1 copy
S445_2	2018							1 copy	1 copy
S445_3	2018							1 copy	1 copy
S446_1	2018	pass	2 copies					⩾2 copies	⩾2 copies
S446_2	2018							⩾2 copies	⩾2 copies

S446_3	2018							ambiguous copy number	⩾2 copies
S447_1	2018	pass	1 copy					1 copy	1 copy
S447_2	2018							1 copy	1 copy
S447_3	2018							1 copy	1 copy
S448_1	2018	pass	1 copy					1 copy	1 copy
S448_2	2018							1 copy	1 copy
S448_3	2018							1 copy	1 copy
S449_1	2018	pass	1 copy					1 copy	1 copy
S449_2	2018							1 copy	1 copy
S449_3	2018							1 copy	1 copy
S450_1	2018	pass	1 copy					1 copy	1 copy
S450_2	2018							1 copy	1 copy
S450_3	2018							1 copy	1 copy
S451_1	2018	pass	1 copy					1 copy	1 copy
S451_2	2018							1 copy	1 copy
S451_3	2018							⩾2 copies	1 copy
S452_1	2018	pass	1 copy					1 copy	1 copy
S452_2	2018							1 copy	1 copy
S452_3	2018							1 copy	1 copy
S453_1	2018	pass	1 copy					1 copy	1 copy
S453_2	2018							1 copy	1 copy
S453_3	2018							1 copy	1 copy
S454_1	2018	pass	ambiguous copy number	pass	1copy			1 copy	1 copy

S454_2	2018							1 copy	1 copy
S454_3	2018							1 copy	1 copy
S455_1	2018	pass	2 copies					⩾2 copies	ambiguous copy number
S455_2	2018							⩾2 copies	1 copy
S455_3	2018							⩾2 copies	⩾2 copies
S456_1	2018	pass	1 copy					1 copy	1 copy
S456_2	2018							1 copy	1 copy
S456_3	2018							1 copy	1 copy
S457_1	2018	pass	ambiguous copy number	pass	2copy			1 copy	⩾2 copies
S457_2	2018							⩾2 copies	⩾2 copies
S457_3	2018							⩾2 copies	⩾2 copies
S458_1	2018	pass	1 copy					1 copy	1 copy
S458_2	2018							1 copy	failed
S458_3	2018							1 copy	failed
S459_1	2018	pass	1 copy					1 copy	1 copy
S459_2	2018							1 copy	1 copy
S459_3	2018							1 copy	1 copy
S460_1	2018	pass	1 copy					1 copy	1 copy
S460_2	2018							1 copy	ambiguous copy number
S460_3	2018							ambiguous copy number	1 copy
S461_1	2018	pass	1 copy					1 copy	1 copy

S461_2	2018							1 copy	1 copy
S461_3	2018							1 copy	1 copy
S462_1	2018	pass	1 copy					1 copy	1 copy
S462_2	2018							1 copy	1 copy
S462_3	2018							1 copy	1 copy
S463_1	2018	pass	2 copies					1 copy	⩾2 copies
S463_2	2018							⩾2 copies	⩾2 copies
S463_3	2018							⩾2 copies	⩾2 copies
S464_1	2018	pass	1 copy					1 copy	1 copy
S464_2	2018							1 copy	1 copy
S464_3	2018							1 copy	1 copy
S465_1	2018	pass	2 copies					⩾2 copies	⩾2 copies
S465_2	2018							⩾2 copies	⩾2 copies
S465_3	2018							⩾2 copies	⩾2 copies
S466_1	2018	pass	2 copies					1 copy	⩾2 copies
S466_2	2018							⩾2 copies	⩾2 copies
S466_3	2018							⩾2 copies	⩾2 copies
S467_1	2018	pass	2 copies					⩾2 copies	⩾2 copies
S467_2	2018							⩾2 copies	⩾2 copies
S467_3	2018							⩾2 copies	⩾2 copies
S468_1	2018	pass	2 copies					1 copy	⩾2 copies
S468_2	2018							⩾2 copies	⩾2 copies
S468_3	2018							⩾2 copies	⩾2 copies
S469_1	2018	pass	2 copies					⩾2 copies	⩾2 copies
S469_2	2018							⩾2 copies	⩾2 copies

S469_3	2018							⩾2 copies	⩾2 copies
S470_1	2018	pass	2 copies					⩾2 copies	⩾2 copies
S470_2	2018							⩾2 copies	⩾2 copies
S470_3	2018							⩾2 copies	⩾2 copies
S471_1	2019	pass	0 copy					0 copy	0 copy
S471_2	2019							0 copy	0 copy
S471_3	2019							0 copy	0 copy
S472_1	2019	pass	0 copy					0 copy	0 copy
S472_2	2019							0 copy	0 copy
S472_3	2019							0 copy	0 copy
S473_1	2019	pass	0 copy					0 copy	0 copy
S473_2	2019							0 copy	0 copy
S473_3	2019							0 copy	0 copy
S474_1	2019	pass	1 copy					1 copy	1 copy
S474_2	2019							1 copy	1 copy
S474_3	2019							1 copy	1 copy
S475_1	2019	pass	1 copy					1 copy	1 copy
S475_2	2019							1 copy	1 copy
S475_3	2019							1 copy	1 copy
S476_1	2019	pass	1 copy					1 copy	1 copy
S476_2	2019							1 copy	1 copy
S476_3	2019							1 copy	1 copy
S477_1	2019	pass	1 copy					1 copy	1 copy
S477_2	2019							1 copy	1 copy
S477_3	2019							1 copy	1 copy

S478_1	2019	pass	1 copy					1 copy	1 copy
S478_2	2019							1 copy	1 copy
S478_3	2019							1 copy	1 copy

"_1": first repetition, "_2": second repetition, "_3": third repetition
